# Empowerment, Resilience, and Burnout Among Nurses With Recovered COVID‐19: A Multicountry Study

**DOI:** 10.1155/jonm/6861497

**Published:** 2025-12-15

**Authors:** Wei-Tien Lee, Hsiang-Chin Hsu, Huan-Fang Lee, Ferry Efendi, Vimala Ramoo, Hsuan-Man Hung

**Affiliations:** ^1^ Department of Nursing, College of Medicine, National Cheng Kung University, Tainan, Taiwan, ncku.edu.tw; ^2^ School of Medicine, College of Medicine, National Cheng Kung University, Tainan, Taiwan, ncku.edu.tw; ^3^ Department of Emergency Medicine, National Cheng Kung University Hospital, College of Medicine, National Cheng Kung University, Tainan, Taiwan, medcol.mw; ^4^ Department of Nursing, Faculty of Nursing, Universitas Airlangga, Surabaya, Indonesia, unair.ac.id; ^5^ School of Nursing and Midwifery, La Trobe University, Melbourne, Australia, latrobe.edu.au; ^6^ Department of Nursing Science, Faculty of Medicine, University Malaya, Kuala Lumpur, Malaysia, um.edu.my; ^7^ Department of Nursing, Fooyin University, Tainan, Taiwan, fy.edu.tw; ^8^ Department of Second-Degree BS in Nursing, Fooyin University, Kaohsiung, Taiwan, fy.edu.tw

## Abstract

**Background:**

The pandemic of COVID‐19 has placed enormous physical and psychological stress on healthcare workers, particularly nurses, resulting in a high level of burnout. To mitigate burnout, nurses who have recovered from COVID‐19 need to be empowered and resilient; however, the relationship between empowerment, resilience, and burnout remains to be determined.

**Aim:**

This study examines the relationships between psychological empowerment, resilience, and burnout in nurses who recovered from COVID‐19 in Taiwan, Malaysia, and Indonesia.

**Designs:**

A cross‐sectional survey study.

**Methods:**

Six hundred five nurses participated in the study, completing online questionnaires that measured psychological empowerment, resilience, and burnout. Data were analyzed using multigroup confirmatory factor analysis and structural equation modeling to assess the relationships between these variables across the three countries.

**Results:**

Indonesian nurses reported the highest levels of resilience and empowerment, while Malaysian nurses had the highest burnout levels. Empowerment and resilience were negatively correlated with burnout across all countries. In Malaysia, psychological empowerment had a direct negative effect on burnout, whereas in Taiwan and Indonesia, the impact of empowerment on burnout was mediated by resilience.

**Conclusions:**

Psychological empowerment and resilience are critical factors in reducing burnout among nurses who have recovered from COVID‐19. Interventions to enhance these factors could help reduce burnout, with strategies tailored to different cultural and healthcare settings.

**Implication for Nursing Management:**

The COVID‐19 pandemic underscores the need for strategies to address nurse burnout. Key approaches include (1) creating a positive work environment by managing workloads, staffing, and resources; (2) enhancing empowerment and resilience through professional development, mentorship, and autonomy‐building initiatives; and (3) implementing resilience‐focused and burnout‐reducing programs, such as mindfulness‐based stress reduction and resilience training.

## 1. Introduction

In the worldwide pandemic response of COVID‐19, healthcare workers have had the burden of both combating the pandemic and proving to be essential frontline supporters [1]. Based on previous research, nurses who are required to contact COVID‐19 patients directly on the front lines have higher rates of infection than other healthcare professionals [2, 3]. The process of caring for COVID‐19 patients has significantly elevated the levels of stress, fatigue, anxiety, and depression of healthcare workers, and these mental health problems become more severe if they contract COVID‐19 themselves [4]. Multinational studies have shown that under the impact of the global COVID‐19 pandemic, nurses face many issues, such as heavy workloads, fear, high stress, anxiety, helplessness, sleep disorders, and depression [5]. The American Nurses Association (ANA) reported that 51% of nurses felt “overwhelmed,” and surveys indicated that 93% of American healthcare workers experienced various levels of stress, with 76% feeling exhausted and burnt out [3]. The lack of nursing personnel increases work burdens, further affecting nurses′ physical and mental states. Under such extreme stress, it has been proven that helping healthcare workers build good resilience and providing positive support can reduce the risk of burnout [6], with psychological empowerment playing a key role. This helps recover from COVID‐19 infection and provides positive motivation for other COVID‐19 patients [7].

Psychological empowerment refers to employees’ perceived control over their work and lives, divided into four dimensions: meaning, competence, self‐determination, and impact [8]. In healthcare, psychological empowerment significantly impacts job satisfaction and organizational commitment [9]. Furthermore, when healthcare workers are given more power and receive good resilience support, it helps prevent adverse effects such as anxiety, burnout, and depression caused by work [10]. This underscores the positive impact of psychological empowerment on individual and organizational levels, highlighting its importance in facing work stress and challenges.

Resilience, the capacity to cope with adversity, adapt to change, maintain functioning, and recover from trauma, is an essential psychological trait [11]. It enables nurses to face difficult situations through resources such as social support, self‐efficacy, work‐life balance, humor, optimism, and realism, thereby preventing psychological distress, improving job satisfaction, and care quality [12, 13]. During the pandemic, nurses’ resilience decreased compared to prepandemic levels [14] and was negatively correlated with fear of infection, resignation intention, burnout, and positive COVID‐19 tests [15, 16]. At the same time, resilience protected against depression, anxiety, and stress [14], moderating the impact of stress on quality of life [17].

Symptoms of burnout (ICD‐11) include feelings of depletion, increasing cynicism and negativity toward one’s work, and a reduction in professional accomplishment [18]. Burnout is divided into three dimensions: emotional exhaustion, depersonalization, and low personal accomplishment [19]. The global prevalence of burnout among nurses was 30.0% over the past 10 years and has especially been getting worse during the COVID‐19 pandemic [20]. The degree of burnout among nurses in Asian countries increased significantly [21] with a rise in moderate to high burnout states [22].

Furthermore, research [23] shows that psychological empowerment can reduce nurses’ burnout by giving them a greater sense of competence and autonomy, improving job satisfaction, and reducing burnout. Additionally, resilience is crucial in dealing with difficult situations and preventing burnout among nurses. The higher the level of resilience, the lower the degree of burnout they experience [24, 25]. Nurses with high resilience can cope more effectively with the challenges and pressures of the pandemic [26], thus reducing the impact of burnout and depression, optimizing their ability to provide quality care, and promoting patient safety and caregiver well‐being [25, 27]. Enhancing nurses’ psychological empowerment and resilience is critical in alleviating their burnout and reducing the negative impacts of the COVID‐19 pandemic on their mental health.

The cultural backgrounds and healthcare systems significantly influence health outcomes during the pandemic. The situation in Asian countries was more critical than in Western countries. Taiwan’s proactive measures helped create a relatively controlled environment during the early stages of the pandemic. In contrast, Indonesia and Malaysia faced higher numbers of COVID‐19 cases and encountered challenges related to healthcare resource allocation and public health management [28].

This study investigates the relationship between burnout, resilience, and psychological empowerment among COVID‐19 survivor nurses in Taiwan, Malaysia, and Indonesia. Additionally, it aims to provide recommendations for healthcare organizations to support and empower their nursing staff effectively during and beyond the pandemic.

Hypothesis.

H1: There is a positive relationship between psychological empowerment and resilience.

H2: There is a negative relationship between both psychological empowerment and resilience with burnout.

H3: There are significant differences in psychological empowerment, resilience, and burnout levels among COVID‐19 survivor nurses across the three countries.

## 2. Methods

### 2.1. Study Design, Setting, and Participants

This research employed a cross‐sectional survey design with convenience sampling. Participants were nurses from Taiwan, Malaysia, and Indonesia who had previously or were currently infected with COVID‐19. Data was collected between October and November 2022 via an online link.

The inclusion criteria were that participants must be nurses diagnosed with COVID‐19. Exclusion criteria included those who had resigned after their COVID‐19 diagnosis. Data was collected through online questionnaires administered outside the participants’ work environment. Based on G‐power software calculations, the required sample size was estimated to be at least 552 nurses across the three countries, with an effect size of 0.15, a confidence level of 95%, a power of 80%, and 29 predictor variables.

### 2.2. Measures

The Psychological Empowerment Scale (PES) was developed by Spreitzer [8] and consists of 12 questions divided into four components. The scale uses a five‐point Likert scale ranging from 1 (*Strongly Disagree*) to 5 (*Strongly Agree*). The score for each subscale is the average of the questions scores, with higher scores indicating higher levels of psychological empowerment. The original scale demonstrated good reliability with a Cronbach’s *α* of 0.72. The Chinese version, translated by Lee et al. [29], also showed good reliability, with Cronbach’s *α* values ranging from 0.72 to 0.86 for the four subscales. In this study, the overall reliability was 0.93, and Cronbach’s *α* values for the subscales ranged from 0.80 to 0.91.

The Connor–Davidson Resilience Scale (CD‐RISC) with 5 components was developed by Connor and Davidson (2003) to measure resilience over the past month. The scale comprises 25 questions scored on a five‐point Likert scale from 0 (Never) to 4 (Always), with total scores ranging from 0 to 100. Higher scores indicate greater resilience; scores above 80 suggest resilience, while scores above 92 indicate high resilience. The original scale had a reliability of 0.89 and a test‐retest reliability of 0.87 [30]. Previous studies [31, 32] used this scale to measure nurses’ resilience, reporting reliability of 0.92–0.93. Concurrent validity ranged from *r* = 0.56 to 0.74. Reliability testing will also be conducted in this study. The original authors facilitated a rigorous translation and back‐translation process by local experts to produce the Chinese version of the 25 questions. In this study, the overall reliability was 0.96, and Cronbach’s *α* values for the subscales ranged from 0.61 to 0.92.

The Copenhagen Burnout Inventory (CBI), developed by Kristensen et al. [33], is an occupational burnout assessment tool comprising 19 questions divided into three components: personal burnout (six questions), work‐related burnout (seven questions), and client‐related burnout (six questions). Personal burnout is the overall physical and psychological fatigue accumulated throughout the day. Work‐related burnout emphasizes the fatigue induced by work, and client‐related burnout highlights fatigue caused by client interaction. Each item is scored on a 0–100 scale: “Never” (0), “Rarely” (25), “Sometimes” (50), “Often” (75), and “Always” (100). Subscale scores are the averages of the item scores, with higher scores indicating higher levels of burnout. The original scale demonstrated good reliability with Cronbach’s *α* values ranging from 0.85 to 0.87 [33]. In this study, the overall reliability was 0.94, and Cronbach’s *α* values for the subscales ranged from 0.84 to 0.91.

### 2.3. Statistical Analysis

The data collected were analyzed using IBM SPSS Statistics 25.0 and Amos 22.0. Descriptive statistics were used to present the demographic distribution and scale scores. Independent *t*‐tests and one‐way ANOVA were conducted to compare differences in scale scores across demographic groups. Pearson’s product‐moment correlation coefficient assessed the relationships between continuous variables. Finally, multigroup analysis via structural equation modeling (SEM) was applied to compare the conceptual relationships between different groups. In the SEM analysis, maximum likelihood estimation (MLE) was adopted, and multigroup analysis was used to compare the differences in path coefficients across countries. Model fit was evaluated using several indices, including the comparative fit index (CFI), root mean square error of approximation (RMSEA), standardized root mean square residual (SRMR), non‐normed fit index (NNFI), and incremental fit index (IFI). Reliability testing was conducted for all scales used in the study [34–37].

### 2.4. Ethics Statement

This study was approved by the National Cheng Kung University Ethics Committee (NCKU HREC‐E‐111‐359‐2).

## 3. Results

### 3.1. Sociodemographic Data and Factors Related to Burnout, Resilience, and Empowerment

Table 1 summarizes the sociodemographic characteristics. The sample consisted of 605 nurses from Taiwan (*n* = 167, 27.6%), Malaysia (*n* = 256, 42.3%), and Indonesia (*n* = 182, 30.1%), with 84.6% being female (*p* < 0.001). The average age of the participants was 34.35 years (SD = 7.71). Educational attainment varied significantly across countries, with a higher proportion of diploma holders in Malaysia (90.2%) compared to Taiwan and Indonesia (*p* < 0.001). Regarding marital status, 66.6% of nurses were married (*p* < 0.001). 85.0% of nurses worked in hospitals (*p* < 0.001). Post‐COVID‐19 physical effects of symptoms were reported by 52.6%, particularly in Malaysia (63.7%, *p* < 0.001), while psychological effects of symptoms were noted by 21.2% (*p* < 0.001).

**Table 1:** Characteristics of sociodemographics.

Variables	Overall (*n* = 605)		Taiwan (*n* = 167)		Malaysia (*n* = 256)		Indonesia (*n* = 182)		*p* value
	*N*	%	*N*	%	*N*	%	*N*	%	
Age (mean ± SD)	34.35 ± 7.71		34.51 ± 8.5		34.65 ± 7.9		33.77 ± 6.6		0.479
Gender									< 0.001^∗^
Male	93	15.4	7	4.2	23	9.0	63	34.6	
Female	512	84.6	160	95.8	233	91.0	119	65.4	
Education									< 0.001^∗^
Diploma	311	51.4	6	3.6	231	90.2	74	40.7	
Bachelor/master/doctoral	294	48.6	161	96.4	25	9.8	108	59.3	
Marital status									< 0.001^∗^
Unmarried	192	31.7	80	47.9	69	27.0	43	23.6	
Married	403	66.6	85	50.9	184	71.9	134	73.6	
Divorced/widowed	10	1.7	2	1.2	3	1.2	5	2.7	
Workplace									< 0.001^∗^
Hospital	514	85.0	159	95.2	250	97.7	105	57.7	
Clinic	28	4.6	2	1.2	6	2.3	20	11.0	
Community/school	63	10.4	6	3.6	0	0.0	57	31.3	
Have you got the physical effects of COVID‐19 symptoms after a negative result test?									< 0.001^∗^
Yes	318	52.6	56	33.5	163	63.7	75	41.2	
No	287	47.4	111	66.5	93	36.3	107	58.8	
Have you got the psychological effects of COVID‐19 symptoms after a negative result test?									< 0.001^∗^
Yes	128	21.2	20	12.0	45	17.6	63	34.6	
No	477	78.8	147	88.0	211	82.4	119	65.4	

^∗^ *p* < 0.05.

Table 2 further illustrates the associations between demographic variables and scores for resilience, empowerment, and burnout. In the overall sample, nurses with diploma degrees had significantly higher resilience and burnout scores. Post hoc Scheffé tests revealed that married nurses had significantly higher scores than unmarried nurses for resilience and empowerment. Nurses working in the community or school had significantly higher resilience and empowerment, while those working in hospitals had significantly higher burnout. Nurses who experienced physical effects of symptoms following a negative COVID‐19 test demonstrated significantly higher burnout scores and lower resilience levels. In Taiwan, female nurses exhibited significantly higher empowerment scores than their male counterparts (*p = *0.04). In Indonesia, the workplace influenced both resilience and burnout, with community or school workers scoring significantly higher on resilience (*p < *0.001) and lower on burnout (*p = *0.013). Across all three countries, nurses who experienced physical or psychological effects of symptoms post‐negative COVID‐19 test had significantly higher burnout scores, except for the physiological effects in Indonesia.

**Table 2:** Comparison of the score of resilience, empowerment, and burnout subscale among demographic variables.

M ± SD	Overall (*n* = 605)			Taiwan (*n* = 167)			Malaysia (*n* = 256)			Indonesia (*n* = 182)		
	Resilience	Empowerment	Burnout	Resilience	Empowerment	Burnout	Resilience	Empowerment	Burnout	Resilience	Empowerment	Burnout
Gender												
Male	2.9 ± 0.7	4.1 ± 1.0	34.5 ± 18.6	2.6 ± 1.2	3.0 ± 1.0	44.7 ± 26.7	3.0 ± 0.5	3.8 ± 1.1	44.2 ± 21.8	2.9 ± 0.6	4.4 ± 0.8	29.8 ± 14.3
Female	2.8 ± 0.6	4.0 ± 0.9	37.2 ± 16.9	2.6 ± 0.6	3.7 ± 0.9	35.5 ± 16.1	2.9 ± 0.6	4.0 ± 1.0	41.2 ± 18.0	2.9 ± 0.5	4.3 ± 0.7	31.9 ± 13.6
*p* value	0.060	0.225	0.157	0.900	0.040^∗^	0.400	0.230	0.250	0.450	0.889	0.582	0.339
Education												
Diploma	2.9 ± 0.6	4.1 ± 0.9	38.9 ± 18.0	2.4 ± 0.8	3.8 ± 1.0	36.8 ± 20.1	2.9 ±.06	4.0 ± 1.0	41.6 ± 18.2	3.0 ± 0.6	4.3 ± 0.8	30.6 ± 14.3
Above bachelor	2.7 ± 0.6	4.0 ± 0.9	34.6 ± 16.1	2.6 ± 0.6	3.7 ± 0.9	35.9 ± 16.6	2.9 ± 0.5	4.3 ± 1.0	40.0 ± 20.2	2.9 ± 0.5	4.4 ± 0.8	31.5 ± 13.6
*p* value	< 0.001^∗∗^	0.209	0.002^∗∗^	0.459	0.752	0.881	0.869	0.206	0.686	0.146	0.573	0.636
Marital status												
Unmarried	2.7 ± 0.7	3.9 ± 0.9	38.9 ± 18.2	2.5 ± 0.6	3.6 ± 0.9	37.9 ± 18.8	2.8 ± 0.6	3.9 ± 1.0	42.8 ± 18.6	2.8 ± 0.7	4.2 ± 0.8	34.5 ± 15.1
Married	2.9 ± 0.6	4.1 ± 0.9	35.8 ± 16.7	2.6 ± 0.6	3.7 ± 0.9	34.1 ± 14.3	2.9 ± 0.6	4.1 ± 1.0	40.9 ± 18.4	3.0 ± 0.5	4.4 ± 0.8	29.9 ± 13.4
Divorced/Widowed	2.9 ± 0.6	4.2 ± 1.1	37.0 ± 12.9	3.1 ± 0.8	3.0 ± 1.4	27 ± 2.8	2.7 ±.0.7	4.2 ± 1.1	44.7 ± 16.8	3.0 ± 0.6	4.6 ± 0.9	36.3 ± 11.6
*p* value	0.004^∗∗^	0.016^∗^	0.120	0.207	0.434	0.266	0.526	0.588	0.731	0.371	0.353	0.108
Workplace												
Hospital	2.8 ± 0.6	4.0 ± 0.9	38.0 ± 17.2	2.6 ± 0.6	3.7 ± 0.9	35.8 ± 16.5	2.9 ± 0.6	4.0 ± 1.0	41.3 ± 18.4	2.9 ± 0.5	4.3 ± 0.7	33.3 ± 13.1
Clinic	2.7 ± 0.7	4.2 ± 0.9	36.3 ± 14.5	2.4 ± 0.7	3.0 ± 1.1	44.1 ± 12.1	3.2 ± 0.6	4.3 ± 0.6	46.3 ± 16.5	2.6 ± 0.7	4.3 ± 0.9	32.5 ± 12.9
Community	3.1 ± 0.6	4.4 ± 0.9	27.4 ± 15.7	2.7 ± 0.7	3.8 ± 1.0	34.0 ± 24.0	0	0	0	3.2 ± 0.6	4.4 ± 0.9	26.7 ± 14.8
*p* value	< 0.001^∗∗^	0.002^∗∗^	< 0.001^∗∗^	0.727	0.567	0.756	0.265	0.532	0.514	< 0.001^∗∗^	0.364	0.013^∗^
Have you got the physical effects of COVID‐19 symptoms after a negative result test?												
Yes	2.8 ± 0.6	4.0 ± 0.9	41.5 ± 16.8	2.5 ± 0.6	3.6 ± 0.9	40.9 ± 16.7	2.9 ± 0.6	3.9 ± 0.9	44.3 ± 18.0	2.9 ± 0.6	4.3 ± 0.8	36.0 ± 12.4
No	2.9 ± 0.6	4.1 ± 0.9	31.6 ± 16.1	2.6 ± 0.7	3.7 ± 0.9	31.2 ± 15.3	3.0 ± 0.6	4.2 ± 1.0	36.4 ± 18.0	3.0 ± 0.6	4.3 ± 0.8	27.7 ± 13.9
*p* value	0.037^∗^	0.096	< 0.001^∗∗^	0.437	0.618	< 0.001^∗∗^	0.059	0.061	0.001^∗∗^	0.132	0.786	0.359
Have you got the psychological effects of COVID‐19 symptoms after a negative result test?												
Yes	2.8 ± 0.6	4.1 ± 1.0	45.2 ± 17.3	2.5 ± 0.7	3.5 ± 1.0	53.3 ± 18.7	2.8 ± 0.5	3.9 ± 1.0	53.8 ± 15.3	2.8 ± 0.6	4.5 ± 0.8	36.5 ± 13.6
No	2.8 ± 0.6	4.0 ± 0.9	34.5 ± 16.5	2.6 ± 0.6	3.7 ± 0.9	33.5 ± 14.9	2.9 ± 0.6	4.1 ± 1.0	38.8 ± 17.9	3.0 ± 0.6	4.3 ± 0.7	28.3 ± 13.2
*p* value	0.360	0.194	< 0.001^∗∗^	0.683	0.491	< 0.001^∗∗^	0.223	0.225	< 0.001^∗∗^	0.119	0.069	< 0.001^∗∗^

^∗^ *p* < 0.05.

^∗∗^ *p* < 0.01.

### 3.2. Comparison of the Scores of Resilience, Empowerment, and Burnout Across Countries

Indonesian nurses exhibited the highest levels of overall resilience (2.93 ± 0.58, *p* < 0.001), as well as all its subdimensions (*p* < 0.001). In contrast, Taiwanese nurses consistently showed the lowest levels of overall resilience (2.58 ± 0.63, *p* < 0.001) and the subdimensions (*p* < 0.001). Similarly, Indonesian nurses demonstrated the highest overall empowerment (4.32 ± 0.78, *p* < 0.001) and the subdimensions (*p* < 0.001), except for the impact dimension (3.92 ± 0.93, *p* < 0.001), whereas Taiwanese nurses reported the lowest overall empowerment (3.66 ± 0.90, *p* < 0.001) and some of the subdimensions. In terms of burnout, Malaysian nurses had the highest levels of overall burnout (41.42 ± 18.39, *p* < 0.001) and all the subdimensions, while Indonesian nurses exhibited the lowest levels of overall burnout (31.14 ± 13.87, *p* < 0.001) and the subdimensions. ANOVA revealed a statistically significant difference among the three countries (*p* = 0.037), indicating that at least one group differs from the others in the population. However, when accounting for multiple comparisons with the Scheffé correction, no single country pair shows a statistically significant difference, although the difference between Malaysia and Indonesia approaches significance (*p* = 0.066) (Table 3).

**Table 3:** Comparison of the score of resilience, empowerment, and burnout subscale in three countries.

Variables	Overall (*n* = 605)	Taiwan (*n* = 167)	Malaysia (*n* = 256)	Indonesia (*n* = 182)	*p* value	Post hoc Scheffé tests
	Mean ± SD	Mean ± SD	Mean ± SD	Mean ± SD		
Resilience	2.82 ± 0.62	2.58 ± 0.63	2.90 ± 0.61	2.93 ± 0.58	< 0.001^∗∗^	I > T; M > T
Personal competence	2.85 ± 0.69	2.59 ± 0.68	2.96 ± 0.68	2.95 ± 0.66	< 0.001^∗∗^	I > T; M > T
Instincts	2.73 ± 0.68	2.53 ± 0.67	2.86 ± 0.68	2.72 ± 0.64	< 0.001^∗∗^	I > T; M > T
Change	2.81 ± 0.60	2.68 ± 0.61	2.77 ± 0.56	2.99 ± 0.62	< 0.001^∗∗^	I > T; I > M
Control	2.88 ± 0.75	2.56 ± 0.79	2.97 ± 0.72	3.06 ± 0.65	< 0.001^∗∗^	I > T; M > T
Spiritual influences	2.98 ± 0.79	2.52 ± 0.70	3.05 ± 0.78	3.30 ± 0.67	< 0.001^∗∗^	I > M > T
Empowerment	4.01 ± 0.93	3.66 ± 0.90	4.02 ± 0.97	4.32 ± 0.78	< 0.001^∗∗^	I > M > T
Meaning	4.31 ± 1.00	3.87 ± 0.93	4.34 ± 1.03	4.68 ± 0.84	< 0.001^∗∗^	I > M > T
Competence	3.92 ± 1.12	3.97 ± 0.99	3.45 ± 1.16	4.54 ± 0.84	< 0.001^∗∗^	I > T > M
Self‐determination	4.00 ± 1.03	3.73 ± 1.02	4.07 ± 1.11	4.14 ± 0.87	< 0.001^∗∗^	I > T; M > T
Impact	3.82 ± 1.23	3.09 ± 1.13	4.22 ± 1.28	3.92 ± 0.93	< 0.001^∗∗^	M > I > T
Burnout	36.79 ± 17.19	35.84 ± 16.65	41.42 ± 18.39	31.14 ± 13.87	< 0.001^∗∗^	M > T > I
Personal	40.06 ± 19.61	38.67 ± 19.83	42.43 ± 21.28	38.00 ± 16.44	0.037^∗^	No significant
Work‐related	38.84 ± 18.14	36.57 ± 18.04	45.33 ± 17.10	31.81 ± 16.56	< 0.001^∗∗^	M> T > I
Client‐related	31.11 ± 20.67	32.16 ± 18.46	35.84 ± 23.06	23.49 ± 16.50	< 0.001^∗∗^	M > I; T > I

Abbreviations: I, Indonesia; M, Malaysia; T, Taiwan.

^∗^ *p* < 0.05.

^∗∗^ *p* < 0.01.

### 3.3. Correlations of Measured Variables

The results of the correlation analysis indicated significant interrelationships among the dimensions of resilience, empowerment, and burnout. Low to moderate positive correlations were observed within the subdimensions of resilience and empowerment. Similarly, low to moderate positive correlations were observed among the burnout subdimensions. Importantly, low to moderate negative correlations were found between resilience and burnout and empowerment and burnout. These findings suggest that higher levels of resilience and empowerment are associated with lower levels of burnout (Appendix Table A1, Table A2, Table A3, and Table A4).

### 3.4. Multigroup Confirmatory Factor Analysis (MCFA) and Multigroup Path Analysis

The MCFA results demonstrated that the model was a good fit. The chi‐square value was 617.817 with 169 degrees of freedom (*p* < 0.001). The CFI was 0.924, the RMSEA was 0.066, within the acceptable range, and the Standardized Root Mean Square Residual (SRMR) was 0.0492, indicating a good fit. The NNFI was 0.911, and the IFI was 0.925. These indices collectively confirm the adequacy of the model fit, supporting the representation of relationships among empowerment, resilience, and burnout in COVID‐19 survivor nurses in different countries (Table 4).

**Table 4:** Goodness‐of‐fit statistics.

**Model-fit index**	**Recommended value**	**Scores**	

Chi‐square/degree of freedom (*χ* ^2^/df) (*p* < 0.001)	≤ 3.00	3.656 (*p* < 0.001)	
Comparative Fit Index (CFI)	≥ 0.90	0.924	
Root mean square error of approximation (RMSEA)	< 0.08	0.066	
Standardized Root Mean square Residual (SRMR)	< 0.08	0.049	
Non‐Normed Fit Index (NNFI)	≥ 0.90	0.911	
Incremental Fit Index (IFI)	≥ 0.90	0.925	

Based on the findings of this study, in Taiwan, empowerment has a moderate positive effect on resilience (*β* = 0.31, *p < *0.001), and resilience exerted a small‐to‐moderate negative effect on burnout (*β* = −0.22, *p = *0.006). However, empowerment’s effect on burnout is nonsignificant (*β* = −0.12, *p = *0.129), indicating that the association was fully mediated by resilience, as evidenced by the significant indirect effect (*p* < 0.01) and the nonsignificant direct effect. In Malaysia, empowerment strongly positively influences resilience (*β* = 0.57, *p < *0.001), and resilience had a small‐to‐moderate negative effect on burnout (*β* = −0.19, *p = *0.005). Furthermore, empowerment significantly reduced burnout with a moderate effect (*β* = −0.32, *p < *0.001). There is an additional indirect effect through resilience (*p < 0.05*), highlighting partial mediation. In Indonesia, empowerment also showed a large effect on resilience (*β* = 0.57, *p < *0.001), and resilience has a moderate‐to‐large negative effect on burnout (*β* = −0.39, *p < *0.001). However, similar to Taiwan, the direct impact of empowerment on burnout is not significant in Indonesia (*β* = −0.09, *p = *0.336), reflecting full mediation through resilience. Overall, the data from these three countries consistently demonstrate that empowerment positively influences resilience (moderate to large effect sizes), and resilience, in turn, negatively affects burnout (small to large effect sizes). Notably, Malaysia was unique in showing both direct and indirect negative effects of empowerment on burnout, which is fully mediated by resilience in Taiwan and Indonesia (Tables 5 and 6).

**Table 5:** Comparison of relationship between empowerment, resilience, and burnout in three countries.

Taiwan	Taiwan		Malaysia		Indonesia		Taiwan versus Malaysia	Taiwan versus Indonesia	Malaysia versus Indonesia
	β	*p*	β	*p*	β	*p*	Z score	Z score	Z score
Empowerment⟶Resilience	0.31	< 0.001	0.57	< 0.001	0.57	< 0.001	2.35^∗^	2.75^∗∗^	−0.70
Resilience⟶Burnout	−0.22	0.006	−0.19	0.005	−0.39	< 0.001	0.27	−1.02	1.37
Empowerment⟶Burnout	−0.12	0.129	−0.32	< 0.001	−0.09	0.34	−1.66	0.36	−2.01^∗^

^∗^ *p* value < 0.05.

^∗∗^ *p* value < 0.01.

**Table 6:** Total effects, direct effects, and indirect effects in three countries.

	Taiwan			Malaysia			Indonesia		
	Total	Direct	Indirect	Total	Direct	Indirect	Total	Direct	Indirect
Empowerment⟶Resilience	0.233^∗∗^	0.233^∗∗^	0.000	0.403^∗∗^	0.403^∗∗^	0.000	0.45^∗^	0.45^∗^	0.000
Resilience⟶Burnout	−5.91^∗^	−5.91^∗^	0.000	−5.14^∗^	−5.14^∗^	0.000	−9.06^∗∗^	−9.06^∗∗^	0.000
Empowerment⟶Burnout	−3.89^∗^	−2.51	−1.379^∗^	−8.13^∗∗^	−6.06^∗∗^	−2.07^∗^	−5.73^∗^	−1.66	−4.07^∗∗^
Mediation	Total			Partial			Total		

^∗^ *p* < 0.05.

^∗∗^ *p* < 0.01.

The z scores were used to indicate where the differences in path strengths between the countries are statistically significant. There are significant differences in the empowerment‐resilience relationship between Taiwan and Malaysia (*z* = 2.348, *p < *0.05) and Taiwan and Indonesia (*z* = 2.745, *p < *0.01). However, there are no significant differences in the resilience‐burnout relationship between the three countries. Notably, the direct effect of empowerment on burnout shows a significant difference between Malaysia and Indonesia (*z* = −2.011, *p < *0.05) (Table 5).

## 4. Discussion

Among nurses who recovered from COVID‐19 in three Asian countries, the study examined the relationship between resilience, empowerment, and burnout. According to the country‐specific findings, Indonesian nurses scored highest in resilience and empowerment compared to Malaysian and Taiwanese nurses. Malaysian nurses scored higher on burnout than Indonesian and Taiwanese nurses. The relationship between resilience and empowerment was positive, while the relationship between resilience and burnout was negative. An analysis of multigroup structural equations confirmed the relationship between empowerment, resilience, and burnout. Across three countries, empowerment and resilience were positively correlated, with significant differences between Taiwan and Malaysia, as well as Taiwan and Indonesia. Malaysia found a partial mediation effect of resilience in the relationship between empowerment and burnout, whereas Taiwan and Indonesia found a total mediation effect of resilience.

### 4.1. The Physical and Psychological Effects of COVID‐19 Symptoms on Empowerment, Resilience, and Burnout

Malaysian nurses have the highest percentage of physical effects of COVID‐19 symptoms. Burnout scores among nurses with physical effects of symptoms were significantly higher in Taiwanese and Malaysian nurses than those without groups, and nurses with psychological effects of COVID‐19 symptoms had significantly higher burnout scores in all three countries.

These findings suggest that the lingering effects of COVID‐19 may function as both physical and psychological stressors that undermine resilience and weaken the protective role of empowerment. Persistent symptoms such as fatigue, dyspnea, or muscle weakness not only reduce work efficiency but also lower nurses’ confidence in their professional competence [38, 39], thereby indirectly increasing their vulnerability to burnout. On the psychological side, anxiety, depression, and post‐traumatic stress may erode nurses’ sense of autonomy and control [38], which are key components of psychological empowerment [8, 9]. This dual burden highlights that post‐COVID‐19 nurses face a compounded risk: diminished physical capacity that heightens exhaustion, combined with psychological distress that weakens coping mechanisms.

Importantly, our findings align with prior studies showing that long COVID symptoms have been associated with poorer self‐rated health, chronic fatigue, and elevated burnout levels among healthcare professionals [38–40]. When empowerment and resilience are compromised by these sequelae, nurses may experience a downward spiral where physical strain and psychological distress reinforce one another. This underscores the necessity for healthcare institutions to provide systematic post‐COVID monitoring, targeted rehabilitation programs, and accessible mental health support [6, 7, 39] to prevent long‐term erosion of resilience and empowerment.

### 4.2. Resilience as a Mediator for the Relationship Between Empowerment and Burnout

Resilience is the ability to successfully manage stressful events, moderating their negative effects and promoting adaptation and psychological well‐being [41]. Resilience partially and totally mediates the relationship between empowerment and burnout in the current study. The findings were also similar to previous studies [38, 42]. Specifically, in Taiwan and Indonesia, empowerment reduced burnout entirely through resilience, suggesting that when resilience is strong, the indirect pathway becomes the dominant mechanism. In contrast, in Malaysia, resilience only partially mediated the relationship, indicating that empowerment retained a direct protective effect on burnout. This highlights cultural and contextual variations in how resilience operates within the empowerment–burnout pathway.

Resilience could partially mediate the relationship between burnout (specifically emotional exhaustion and depersonalization) and psychological distress [42], and resilience is a crucial factor in maintaining a healthy psychological state, regardless of burnout. Thus, resilience provides a foundation for effectively applying coping strategies when faced with psychological distress. Prior studies confirm that nurses with higher resilience are more likely to use adaptive coping strategies, including problem‐focused and emotion‐focused coping, which directly reduce burnout and enhance recovery [43, 44]. Moreover, resilience influences how stressors are appraised, which in turn determines the effectiveness of coping strategies [41]. This mechanism explains why resilience can buffer against the erosion of empowerment under extreme workplace demands.

Our findings further support the notion that resilience not only protects against immediate stress but also sustains empowerment by reinforcing self‐determination, competence, and meaning [8, 9, 24]. In this sense, resilience functions as both a shield and a bridge—shielding nurses from the detrimental effects of burnout while bridging the empowering aspects of their role to psychological well‐being. Therefore, resilience‐building programs, such as mindfulness‐based training and structured peer support, should be prioritized as part of workforce development strategies [11]. Such interventions would not only reduce burnout but also amplify the positive effects of empowerment in different healthcare contexts.

### 4.3. Cultural and Healthcare Contextual Differences

The cross‐country differences observed in this study highlight the role of cultural and healthcare system contexts. The influence of empowerment on reliance was significant in these countries, but Taiwan differed from Malaysia and Indonesia, and the two countries were similar in the current study.

Cultural factors such as collectivism versus individualism may play a role in these differences [45]. Collectivism values group harmony and collective responsibility, while individualism values individual autonomy and self‐expression [46, 47]. These values can shape how people view risk and reward and make decisions [46, 47]. According to previous research, Malaysia and Indonesia tend to be collectivist, and Taiwan combines collectivism and individualism [45]. In strongly collectivist contexts such as Malaysia and Indonesia, empowerment is often expressed through shared goals, mutual support, and spiritual practices. Nurses gain resilience not only from their personal capacities but also from social networks, family ties, and communal values [48]. This cultural embedding amplifies the effect of empowerment on resilience because the collective setting reinforces individual confidence and coping capacity.

By contrast, Taiwan reflects a hybrid cultural orientation where collectivism coexists with elements of individualism. In Taiwanese healthcare settings, nurses may place greater emphasis on personal responsibility, autonomy, and self‐efficacy [49]. This balance may weaken the direct translation of empowerment into resilience compared with the stronger collectivist environments of Malaysia and Indonesia. In other words, empowerment in Taiwan may require more individualized strategies—such as fostering professional autonomy and encouraging self‐reflection—to achieve the same level of resilience gains.

Healthcare system differences may also contribute. Taiwan’s proactive pandemic measures created a relatively controlled environment during the early phase of COVID‐19 [28]. While this limited case burden may have buffered stress, it may also have reduced the urgency for empowerment‐driven resilience compared with Malaysia and Indonesia, where higher case numbers and resource shortages forced nurses to rely more heavily on empowerment and resilience to cope. This suggests that structural healthcare conditions—such as staffing adequacy, resource distribution, and policy responses—can shape the psychological mechanisms linking empowerment, resilience, and burnout.

Taken together, these findings underscore the need for culturally sensitive and context‐specific interventions. In collectivist societies, strengthening social support systems, teamwork, and spiritual resources may be especially effective, while in more individualist‐leaning contexts, strategies that emphasize autonomy, self‐efficacy, and personal growth may better reinforce resilience and reduce burnout.

### 4.4. Strengths and Limitations

The study’s strength was that multicountry survey studies provide a robust framework for understanding global dynamics through diverse perspectives, enhanced sample sizes, comparative insights, and localized knowledge. These strengths are essential for organizations seeking to navigate the complexities of international markets effectively. In addition, the SEM multigroup analysis offers significant strengths in examining group differences and uncovering mediation effects into complex relationships among variables [34–37]. These advantages make it a valuable tool for researchers to understand nuanced dynamics within diverse populations.

However, the current study had some limitations. One of the primary limitations of using SEM in cross‐sectional studies is the difficulty in establishing causal relationships. The data collection method focuses on some areas, and the participant’s representative must consider it. As the study employed convenience sampling and voluntary participation through online surveys, selection bias cannot be completely ruled out. Although inclusion/exclusion criteria were applied and multicountry recruitment increased sample diversity, the representativeness of the sample may still be limited. Finally, respondents may provide biased or subjective answers influenced by their current mood, recent experiences, or social desirability pressures.

## 5. Conclusions

The study highlights the crucial role of resilience and empowerment in reducing burnout among nurses in Asia. While resilience partially mediated the relationship between empowerment and burnout in Malaysia, it served as a total mediator in Taiwan and Indonesia, underscoring the importance of fostering resilience to mitigate burnout across different cultural contexts. These findings suggest targeted interventions to enhance resilience and empowerment could be beneficial in decreasing nurses’ burnout.

## 6. Implications for Nursing Management

The COVID‐19 pandemic has underscored the critical need for effective management strategies to address nurse burnout, given its significant impact on nurses’ mental health and work capacity. Managers can adopt some strategies: (1) create a positive work environment and (2) provide interventions related to improving empowerment and resilience and decreasing burnout.

### 6.1. Creative a Positive Work Environment

Effective management of nurse burnout requires careful attention to workload and resource allocation. Studies have shown that increased workloads, lack of staffing, and inadequate resources are major contributors to burnout among nurses. A systematic review and meta‐analysis highlighted that working in high‐risk environments, longer working times in quarantine areas, and inadequate material and human resources significantly increased burnout levels among nurses [50]. Healthcare organizations should prioritize removing barriers to nurses’ workflow and addressing workplace environment concerns to decrease the rate of burnout. This includes ensuring adequate staffing, providing necessary resources for patient care, and implementing flexible scheduling to reduce the strain on nurses. Increasing personal protective equipment, providing an alternative workforce, and periodic rotation of front‐line nurses can also help prevent or reduce burnout [51].

### 6.2. Provide the Interventions Related to Improving Empowerment and Resilience as Well as Decreasing Burnout

#### 6.2.1. Strategies Related to Improving Empowerment

Empowerment is a critical factor for improving resilience and mitigating burnout among nurses. Effective strategies to improve psychological empowerment among nurses should encompass opportunities for meaningful engagement, skill enhancement, autonomy, and recognition. Firstly, providing nurses with opportunities to observe the impact of their work—such as through patient feedback sessions and acknowledgment of their unique contributions via regular recognition or awards programs—strengthens their sense of purpose and perception of meaningful work. Continuous professional development and mentorship are essential, as they cultivate competence and self‐efficacy, equipping nurses to manage complex care scenarios with greater confidence. Additionally, continuous professional development and mentorship are essential, as they cultivate competence and self‐efficacy, equipping nurses to manage complex care scenarios with greater confidence and directly boosting job satisfaction [52, 53].

#### 6.2.2. Strategies Related to Improving Resilience

Managers should prioritize evidence‐based resilience training programs, such as the SMART program and mindfulness‐based interventions, which have been shown to improve resilience among health professionals significantly. Training in mindfulness, cognitive behavioral therapy, and stress management led to positive resilience outcomes, which improved staff retention and well‐being. Second, sustainable resilience requires addressing systemic workplace stressors. Managers can promote resilience by offering flexible work hours and peer support programs. Integrating these practices can create a work culture that values self‐care, reduces turnover, and enhances job satisfaction. Third, it is imperative to encourage self‐reflection and self‐care initiatives to maintain resilience. Health professionals can manage work‐related stress more effectively if provided with structured opportunities for reflection, mentorship, and personal development [11].

#### 6.2.3. Strategies Related to Decreasing Burnout

Implementing psychological support and self‐care programs is crucial in mitigating burnout. An umbrella review evaluated individual‐based evidence to reduce nurse burnout, which included mental health, physical activity, and professional competence strategies. The most effective interventions were MBSR, resilience and cognition training, stress and relaxation, and yoga for occupational stress. Regular assessments, tailored schedules, well‐trained instructors, resilience improvement, professional value education, continuing education opportunities, peer support, and a supervision system should be implemented [54].

## Conflicts of Interest

The authors declare no conflicts of interest.

## Author Contributions

Huan‐Fang Lee: conceptualization, funding acquisition, project administration, supervision. Ferry Efendi, Vimala Ramoo, Hsuan‐Man Hung: data curation. Wei‐Tien Lee, Hsiang‐Chin Hsu: formal analysis. Ferry Efendi, Vimala Ramoo: resources. Wei‐Tien Lee, Hsiang‐Chin Hsu, Huan‐Fang Lee: validation, visualization, writing–original draft. Hsiang‐Chin Hsu, Huan‐Fang Lee: writing–review and editing. Hsiang‐Chin Hsu and Wei‐Tien Lee contributed equally to this work. Hsiang‐Chin Hsu has the same contribution as Wei‐Tien Lee.

## Funding

This research was partly supported by the Higher Education Sprout Project, Ministry of Education to the Headquarters of University Advancement at National Cheng Kung University (NCKU D111‐B5524).

## Data Availability

Data are available on request.

## Appendix A

**Table A1:** Correlation between variables for 3 countries.

Variables	Age	R_Average	R_Personal competence	R_Instincts	R_Change	R_Control	R_Spiritual	E_Average	E_Meaning	E_Competence	E_Self‐Deter	E_Impact	B_Average	B_Personal	B_Work
R_Average	0.085^∗^														
R_Personal competence	0.069	0.961^∗∗^													
R_Instincts	0.077	0.935^∗∗^	0.877^∗∗^												
R_Change	0.084^∗^	0.858^∗∗^	0.757^∗∗^	0.722^∗∗^											
R_Control	0.090^∗^	0.901^∗∗^	0.858^∗∗^	0.802^∗∗^	0.728^∗∗^										
R_Spiritual	0.069	0.735^∗∗^	0.645^∗∗^	0.598^∗∗^	0.664^∗∗^	0.636^∗∗^									
E_ Average	0.080^∗^	0.500^∗∗^	0.506^∗∗^	0.425^∗∗^	0.433^∗∗^	0.480^∗∗^	0.353^∗∗^								
E_Meaning	0.090^∗^	0.446^∗∗^	0.454^∗∗^	0.349^∗∗^	0.390^∗∗^	0.442^∗∗^	0.370^∗∗^	0.905^∗∗^							
E_Competence	0.049	0.382^∗∗^	0.366^∗∗^	0.267^∗∗^	0.430^∗∗^	0.367^∗∗^	0.331^∗∗^	0.797^∗∗^	0.732^∗∗^						
E_Self‐Deter	0.041	0.429^∗∗^	0.443^∗∗^	0.384^∗∗^	0.344^∗∗^	0.411^∗∗^	0.264^∗∗^	0.919^∗∗^	0.772^∗∗^	0.671^∗∗^					
E_Impact	0.091^∗^	0.443^∗∗^	0.459^∗∗^	0.438^∗∗^	0.314^∗∗^	0.413^∗∗^	0.245^∗∗^	0.793^∗∗^	0.613^∗∗^	0.342^∗∗^	0.704^∗∗^				
B_ Average	−0.108^∗∗^	−0.323^∗∗^	−0.332^∗∗^	−0.281^∗∗^	−0.281	−0.309^∗∗^	−0.194^∗∗^	−0.327^∗∗^	−0.300^∗∗^	−0.320^∗∗^	−0.273^∗∗^	−0.224^∗∗^			
B_Personal	−0.085^∗^	−0.299^∗∗^	−0.299^∗∗^	−0.283^∗∗^	−0.260^∗∗^	−0.286^∗∗^	−0.141^∗∗^	−0.275^∗∗^	−0.247^∗∗^	−0.224^∗∗^	−0.242^∗∗^	−0.223^∗∗^	0.869^∗∗^		
B_Work	−0.086^∗^	−0.290^∗∗^	−0.294^∗∗^	−0.248^∗∗^	−0.280^∗∗^	−0.265^∗∗^	−0.160^∗∗^	−0.316^∗∗^	−0.279^∗∗^	−0.318^∗∗^	−0.270^∗∗^	−0.214^∗∗^	0.938^∗∗^	0.781^∗∗^	
B_Client	−0.115^∗∗^	−0.271^∗∗^	−0.288^∗∗^	−0.218^∗∗^	−0.208^∗∗^	−0.271^∗∗^	−0.214^∗∗^	−0.276^∗∗^	−0.270^∗∗^	−0.305^∗∗^	−0.212^∗∗^	−0.159^∗∗^	0.849^∗∗^	0.540^∗∗^	0.706^∗∗^

Abbreviations: B, burnout; E, empowerment, R, resilience.

^∗^ *p* value < 0.05.

^∗∗^ *p* value < 0.01.

**Table A2:** Correlation between variables for Taiwan.

Variables	Age	R_Average	R_Personal competence	R_Instincts	R_Change	R_Control	R_Spiritual	E_Average	E_Meaning	E_Competence	E_Self‐Deter	E_Impact	B_Average	B_Personal	B_Work
R_Average	0.100														
R_Personal competence	0.063	0.958^∗∗^													
R_Instincts	0.958^∗∗^	0.952^∗∗^	0.880^∗∗^												
R_Change	0.134	0.887^∗∗^	0.781^∗∗^	0.801^∗∗^											
R_Control	0.177^∗^	0.920^∗∗^	0.875^∗∗^	0.843^∗∗^	0.779^∗∗^										
R_Spiritual	0.048	0.770^∗∗^	0.685^∗∗^	0.693^∗∗^	0.691^∗∗^	0.662^∗∗^									
E_ Average	.0011	0.306^∗∗^	0.265^∗∗^	0.284^∗∗^	0.291^∗∗^	0.361^∗∗^	0.196^∗^								
E_Meaning	0	0.238^∗∗^	0.214^∗∗^	0.199^∗^	0.229^∗∗^	0.297^∗∗^	0.162^∗^	0.917^∗∗^							
E_Competence	−0.007	0.290^∗∗^	0.240^∗∗^	0.271^∗∗^	0.312^∗∗^	0.300^∗∗^	0.213^∗∗^	0.874^∗∗^	0.824^∗∗^						
E_Self‐Deter	−0.026	0.291^∗∗^	0.271	0.259^∗∗^	0.261^∗∗^	0.345^∗∗^	0.176^∗^	0.930^∗∗^	0.827^∗∗^	0.768^∗∗^					
E_Impact	0.064	0.264^∗∗^	0.215^∗∗^	0.270^∗∗^	0.232^∗∗^	0.332^∗∗^	0.148	0.829^∗∗^	0.636^∗∗^	0.543^∗∗^	0.712^∗∗^				
B_ Average	0.025	−0.260^∗∗^	−0.248^∗∗^	−0.208^∗∗^	−0.273^∗∗^	−0.309^∗∗^	−0.124	−0.167^∗^	−0.167^∗^	−0.061	−0.175^∗^	−0.182^∗^			
B_Personal	0.086	−0.215^∗∗^	−0.184^∗^	−0.202^∗∗^	−0.224^∗∗^	−0.263^∗∗^	−0.082	−0.152	−0.133	−0.044	−0.156^∗^	−0.194^∗^	0.890^∗∗^		
B_Work	0.026	−0.250^∗∗^	−0.236^∗∗^	−0.203^∗∗^	−0.267^∗∗^	−0.299^∗∗^	−0.114	−0.199^∗∗^	−0.184^∗^	−0.072	−0.212^∗∗^	−0.228^∗∗^	0.956^∗∗^	0.848^∗∗^	
B_Client	−0.052	−0.226^∗∗^	−0.243^∗∗^	−0.146	−0.234^∗∗^	−0.260^∗∗^	−0.136	−0.087	−0.125	−0.046	−0.091	−0.052	0.810^∗∗^	0.501^∗∗^	0.679^∗∗^

Abbreviations: B, burnout; E, empowerment; R, resilience.

^∗^ *p* value < 0.05.

^∗∗^ *p* value < 0.01.

**Table A3:** Correlation between variables for Malaysia.

Variables	Age	R_Average	R_Personal competence	R_Instincts	R_Change	R_Control	R_Spiritual	E_Average	E_Meaning	E_Competence	E_Self‐Deter	E_Impact	B_Average	B_Personal	B_Work
R_Average	0.155^∗^														
R_Personal competence	0.167^∗∗^	0.959^∗∗^													
R_Instincts	0.154^∗^	0.950^∗∗^	0.896^∗∗^												
R_Change	0.102	0.828^∗∗^	0.715^∗∗^	0.706^∗∗^											
R_Control	0.109	0.884^∗∗^	0.836^∗∗^	0.815^∗∗^	0.665^∗∗^										
R_Spiritual	0.116	0.720^∗∗^	0.613^∗∗^	0.610^∗∗^	0.649^∗∗^	0.575^∗∗^									
E_ Average	0.239^∗∗^	0.551^∗∗^	0.595^∗∗^	0.502^∗∗^	0.410^∗∗^	0.485^∗∗^	0.314^∗∗^								
E_Meaning	0.240^∗∗^	0.460^∗∗^	0.497^∗∗^	0.401^∗∗^	0.333^∗∗^	0.424^∗∗^	0.311^∗∗^	0.885^∗∗^							
E_Competence	0.194^∗∗^	0.471^∗∗^	0.487^∗∗^	0.371^∗∗^	0.411^∗∗^	0.425^∗∗^	0.401^∗∗^	0.816^∗∗^	0.727^∗∗^						
E_Self‐Deter	0.180^∗∗^	0.460^∗∗^	0.504^∗∗^	0.420^∗∗^	0.331^∗∗^	0.423^∗∗^	0.233^∗∗^	0.933^∗∗^	0.772^∗∗^	0.731^∗∗^					
E_Impact	0.201^∗∗^	0.478^∗∗^	0.531^∗∗^	0.503^∗∗^	0.319^∗∗^	0.378^∗∗^	0.139^∗^	0.776^∗∗^	0.556^∗∗^	0.352^∗∗^	0.682^∗∗^				
B_ Average	−0.257^∗∗^	−0.371^∗∗^	−0.421^∗∗^	−0.385^∗∗^	−0.153^∗^	−0.326^∗∗^	−0.220^∗∗^	−0.430^∗∗^	−0.358^∗∗^	−0.334^∗∗^	−0.379^∗∗^	−0.389^∗∗^			
B_Personal	−0.234^∗∗^	−0.340^∗∗^	−0.368^∗∗^	−0.347^∗∗^	−0.184^∗∗^	−0.309^∗∗^	−0.188^∗∗^	−0.356^∗∗^	−0.316^∗∗^	−0.265^∗∗^	−0.291^∗∗^	−0.334^∗∗^	0.893^∗∗^		
B_Work	−0.249^∗∗^	−0.369^∗∗^	−0.416^∗∗^	−0.383^∗∗^	−0.176^∗∗^	−0.306^∗∗^	−.213^∗∗^	−0.446^∗∗^	−0.351^∗∗^	−0.308^∗∗^	−0.413^∗∗^	−0.435^∗∗^	0.948^∗∗^	0.833^∗∗^	
B_Client	−0.218^∗∗^	−0.303^∗∗^	−0.363^∗∗^	−0.321^∗∗^	−0.064	−0.275^∗∗^	−0.196^∗∗^	−0.373^∗∗^	−0.307^∗∗^	−0.333^∗∗^	−0.332^∗∗^	−0.296^∗∗^	0.880^∗∗^	0.612^∗∗^	0.760^∗∗^

Abbreviations: B, burnout; E, empowerment; R, resilience.

^∗^ *p* value < 0.05.

^∗∗^ *p* value < 0.01.

**Table A4:** Correlation between variables for Indonesia.

Variables	Age	R_Average	R_Personal competence	R_Instincts	R_Change	R_Control	R_Spiritual	E_Average	E_Meaning	E_Competence	E_Self‐Deter	E_Impact	B_Average	B_Personal	B_Work
R_Average	−0.028														
R_Personal competence	−0.073	0.961^∗∗^													
R_Instincts	−0.051	0.914^∗∗^	0.841^∗∗^												
R_Change	0.044	0.896^∗∗^	0.814^∗∗^	0.737^∗∗^											
R_Control	−0.024	0.887^∗∗^	0.849^∗∗^	0.748^∗∗^	0.763^∗∗^										
R_Spiritual	0.079	0.675^∗∗^	0.586^∗∗^	0.501^∗∗^	0.655^∗∗^	0.583^∗∗^									
E_ Average	−0.099	0.523^∗∗^	0.526^∗∗^	0.420^∗∗^	0.524^∗∗^	0.475^∗∗^	0.317^∗∗^								
E_Meaning	−0.029	0.504^∗∗^	0.510^∗∗^	0.365^∗∗^	0.522^∗∗^	0.464^∗∗^	0.372^∗∗^	0.903^∗∗^							
E_Competence	−.091	0.520^∗∗^	0.513^∗∗^	0.375^∗∗^	0.561^∗∗^	0.502^∗∗^	0.352^∗∗^	0.907^∗∗^	0.880^∗∗^						
E_Self‐Deter	−.143	0.445^∗∗^	0.447^∗∗^	0.402^∗∗^	0.409^∗∗^	0.368^∗∗^	0.251^∗∗^	0.894	0.705^∗∗^	0.714^∗∗^					
E_Impact	−.090	0.409^∗∗^	0.416^∗∗^	0.359^∗∗^	0.390^∗∗^	0.372^∗∗^	0.171^∗^	0.874^∗∗^	0.661^∗∗^	0.668^∗∗^	0.776^∗∗^				
B_ Average	−0.037	−0.412^∗∗^	−0.392^∗∗^	−0.346^∗∗^	−0.446^∗∗^	−0.363^∗∗^	−0.220^∗∗^	−0.268^∗∗^	−0.316^∗∗^	−0.257^∗∗^	−0.198^∗∗^	−0.192^∗∗^			
B_Personal	−0.027	−0.408^∗∗^	−0.396^∗∗^	−0.354^∗∗^	−0.426^∗∗^	−0.364^∗∗^	−0.178^∗^	−0.290^∗∗^	−0.288^∗∗^	−0.256^∗∗^	−0.282^∗∗^	−0.213^∗∗^	0.820^∗∗^		
B_Work	−0.027	−0.350^∗∗^	−0.343^∗∗^	−0.310^∗∗^	−0.374^∗∗^	−0.277^∗∗^	0.147^∗^	−0.235^∗∗^	−0.277^∗∗^	−0.218^∗∗^	−0.170^∗^	−0.178^∗^	0.908^∗∗^	0.671^∗∗^	
B_Client	−0.04	−0.280^∗∗^	−0.246^∗∗^	−0.206^∗∗^	−0.325^∗∗^	−0.278^∗∗^	−0.237^∗∗^	−0.149^∗^	−0.229^∗∗^	−0.173^∗^	−0.046	−0.091	0.780^∗∗^	0.401^∗∗^	0.578^∗∗^

Abbreviations: B, burnout; E, empowerment; R, resilience.

^∗^ *p* value < 0.05.

^∗∗^ *p* value < 0.01.
